# A comparison of the infant gut microbiome before versus after the start of the covid-19 pandemic

**DOI:** 10.1038/s41598-023-40102-y

**Published:** 2023-08-16

**Authors:** Francesca R. Querdasi, Sarah C. Vogel, Moriah E. Thomason, Bridget L. Callaghan, Natalie H. Brito

**Affiliations:** 1 University of California, Los Angeles, Los Angeles, USA; 2 New York University, New York, USA; 3 New York University Langone Medical Center, New York, USA

**Keywords:** Psychology, Microbiology

## Abstract

The COVID-19 pandemic and resulting public health directives led to many changes in families’ social and material environments. Prior research suggests that these changes are likely to impact composition of the gut microbiome, particularly during early childhood when the gut microbiome is developing most rapidly. Importantly, disruption to the gut microbiome during this sensitive period can have potentially long-lasting impacts on health and development. In the current study, we compare gut microbiome composition among a socioeconomically and racially diverse group of 12-month old infants living in New York City who provided stool samples before the pandemic (N = 34) to a group who provided samples during the first 9-months of the pandemic (March–December 2020; N = 20). We found that infants sampled during the pandemic had lower alpha diversity of the microbiome, lower abundance of Pasteurellaceae and *Haemophilus*, and significantly different beta diversity based on unweighted Unifrac distance than infants sampled before the pandemic. Exploratory analyses suggest that gut microbiome changes due to the pandemic occurred relatively quickly after the start of the pandemic and were sustained. Our results provide evidence that pandemic-related environmental disruptions had an impact on community-level taxonomic diversity of the developing gut microbiome, as well as abundance of specific members of the gut bacterial community.

## Introduction

The start of the COVID-19 pandemic in early 2020 marked a series of drastic changes to the social and material environments of children and families in the United States. Among these were the closure of schools and childcare centers, fewer trips outside the home, reduced access to outdoor spaces, changes in pollutant exposure due to lower levels of air and land vehicular traffic and increased time spent indoors, decreased socialization with people outside immediate households, changes in hygiene habits, and shifts in caregiver mental health and family functioning^1^. Though not yet characterized, there has been speculation that the pandemic could have had a drastic impact on children’s physiology, particularly the development of the gut microbiome^2^. In early life, the gut microbiome has been shown to be strongly influenced by aspects of the social and physical environment that were altered with the onset of the COVID-19 pandemic, including hygiene practices, daycare attendance, nutrition practices, and caregiver stress, to name a few^3–8^. Changes to gut microbiome composition and diversity during this period have been associated with physical and mental health outcomes across the lifespan^4,9–13^. As such, understanding how pandemic-related environmental changes influenced infants’ gut microbiome is important for discerning the potential broader impacts of the pandemic on children’s development.

Rapid colonization of the gut by microbes occurs during and immediately following birth and continues throughout early life^14–21^. The first year of life is typically characterized by massive increases in within-individual bacterial diversity (alpha diversity) in the gut^18^. During this sensitive period of development there are a number of social and environmental factors that influence the gut microbiome^8,18,19,22–25^. Specifically, breastfeeding has shown to be associated with lower alpha diversity of the gut microbiome, and across studies there is evidence that breastfed infants generally have a higher abundance of *Bifidobacterium* and decreased abundance of *Bacteroides* and *Firmicutes* compared to non-breastfed infants^25^. Attendance at daycare has also shown to be associated with reduced abundance of bacteria from the Bifidobacteriaceae, Lactobacillaceae, Staphylococcaceae, and Pasteurellaceae families and higher abundance from the Prevotellaceae, Lachnospiraceae, and Ruminococcaceae families^8^. Though directionality has differed, studies of early life stress and the gut microbiome have found stress to be associated with differences in the relative abundances of several taxa of bacteria, including bacteria from the Lachnospiraceae, Bifidobacteraceae, and Tannerelaceae families^3,4^. These social influences on the early gut microbiome may all have been affected by the social changes caused by the COVID-19 pandemic, with infants potentially experiencing more time at home, less time in daycare interacting with other children, increased hygiene in the environment, changes to diet and breastfeeding practices, and increased caregiver stress all as a result of the pandemic. Altogether, available evidence suggests that the social upheavals caused by the COVID-19 pandemic and related changes in lifestyle for children and families could produce significant changes in the composition of the infant gut microbiome.

Initial evidence from a single study in adults substantiates the possibility that environmental changes associated with pandemic public health directives may have altered the human gut microbiome^26^. Specifically, a study in adults reported decreased abundance of bacteria from the phylum Actinobacteria and increased abundance of bacteria from the phylum Bacteroidetes in those tested early in the pandemic compared to before the pandemic. These findings provide support for the hypothesis that the COVID-19 pandemic and coincident changes to the social and physical environment shaped individual gut microbiome composition. However, given the distinct composition of the gut microbiome in infancy and the particularly high malleability of the gut microbiome in early life, it is possible that pandemic-related microbiome impacts may be different in infants than that reported in adults.

**Figure 1:**
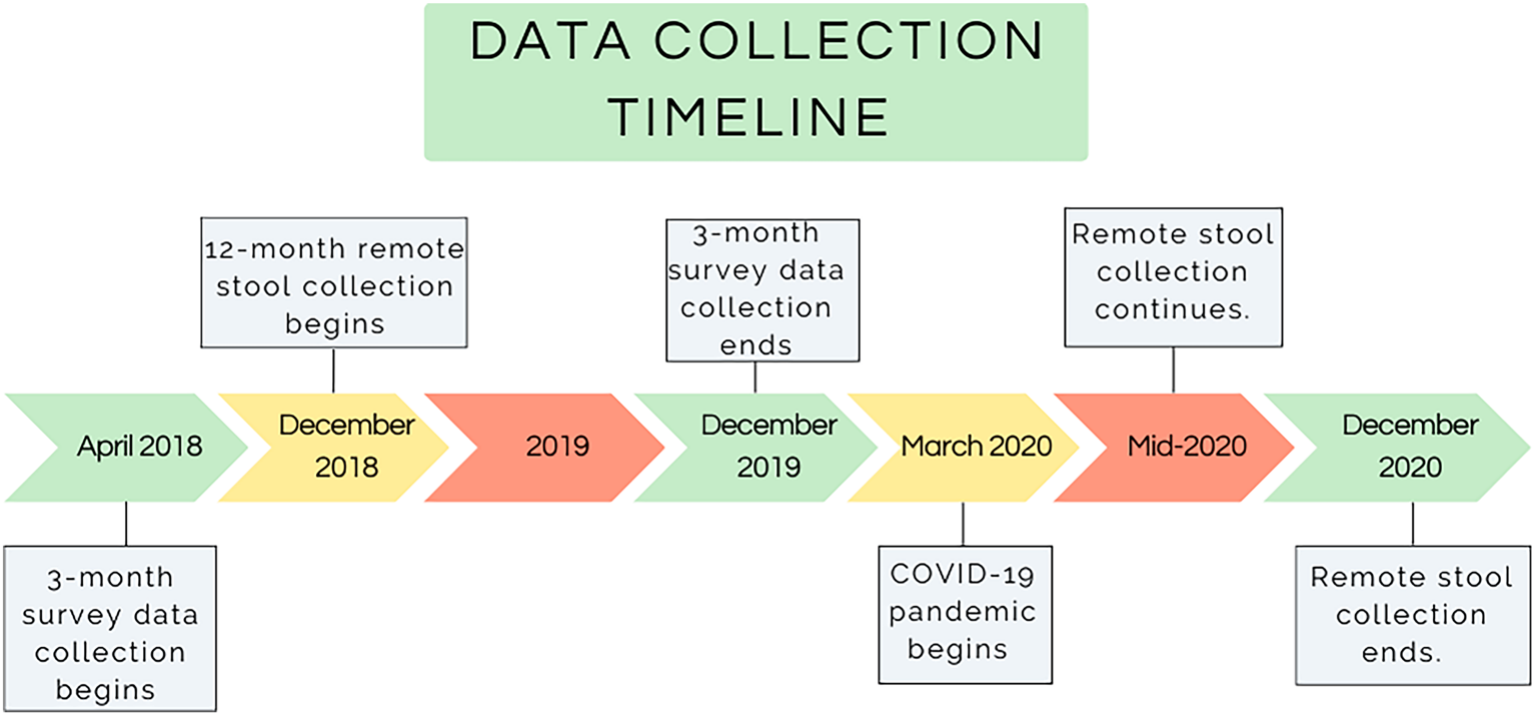
Timeline of data collection for this longitudinal study. Remote stool collection began in December 2018 when the first infants turned 12 months old. The COVID-19 pandemic began in March 2020, with New York State Executive Order 202.8 going into effect on March 22, 2020. Remote stool collection continued throughout the pandemic, and ended in December 2020 when the last infant in the study turned 12 months.

In the current study, we use microbiome data collected from infants between December 2018 and December 2020. See Fig. 1 for a timeline of data collection. The onset of the COVID-19 pandemic in March 2020 renders this a natural experiment by which to assess compositional differences in infant gut microbiome between those who provided a sample before versus after the start of the pandemic. Due to widespread reductions in social activity outside the home, increased hand hygiene, and changes in patterns of family interactions brought on by COVID-19 public health directives, we hypothesized that samples collected after the start of the pandemic would have significantly lower alpha diversity than pre-pandemic samples. Drawing from previous studies documenting the effects of social behaviors on the developing gut microbiome, including those reviewed above, we also hypothesized differences between groups in the relative abundance of bacteria from families found in at least two studies in a targeted analysis: Bifidobacteriaceae, Lactobacillaceae, Pasteurellaceae, Lachnospiraceae, Ruminococcaceae, Tannerellaceae, Bacteroidaceae, and Streptococcaceae^3–8,26^. We also explored group differences on the more detailed genus and species levels, examining all taxa within the gut microbiome in an exploratory analysis with no specific hypotheses about individual taxa. Finally, we hypothesized that, in the pandemic group, the effects of the pandemic on the gut microbiome would be stronger with more time spent in the pandemic, such that alpha diversity would be negatively associated with collection date (i.e., lower towards the end of 2020), and taxa associated with pandemic group membership would have a negative relationship with collection date.

## Results

### Participant characteristics

The racially and socioeconomically diverse sample consisted of 54 infants and their primary caregivers, Table 1.

**Table 1:** Characteristics of sample and sample comparisons.

Variable	Pre-Pandemic Group (N = 34)		Pandemic Group (N = 20)		t-value	*P*-value
	Mean (SD)	Range (sample min–max)	Mean (SD)	Range (sample min–max)		
**Socioeconomic status**						
Income-to-needs ratio	5.94 (6.34)	0.09–24.06	7.69 (6.75)	0.22–23.44	− 0.89	.38
Maternal education (years)	15.88 (2.75)	10.5–20	17.05 (4.29)	8–24	− 1.09	.28
Paternal education (years)	15.38 (3.18)	12–25	15.16 (3.92)	6–20	0.21	.84
Material hardship	2.1 (2.14)	0–7	1.1 (1.89)	0–7	1.74	.09
**Caregiver mental health**						
Perceived stress (3 months)	13.47 (6.35)	0–28	12.35 (5.73)	6–26	0.64	.52
Postpartum depression (3 months)	5.1 (4.12)	0–14	5.7 (5.1)	0–15	− 0.44	.66
State anxiety (3 months)	33.6 (9.62)	20–51	30.35 (9.75)	20–55	1.16	.25
Trait anxiety (3 months)	35.17 (10.25)	20–57	32.45 (11.26)	20–63	0.87	.39
Perceived stress (12 months)	11.29 (6.13)	1–24	14.25 (6.77)	3–25	− 1.58	.12
Depression (12 months)	4.73 (4.66)	0–21	5.35 (5.84)	0–24	− 0.40	.69
**Infant diet and lifestyle**						
Average daily carbohydrates (g)	79.68 (31.44)	17–157.5	82.77 (19.88)	54.5–126.5	− 0.38	.71
Average daily proteins (g)	29.05 (12.13)	9–60.5	28.38 (9.62)	16–43	0.19	.85
Average daily fats (g)	22.84 (10.3)	9.5–41	30.31 (11.16)	14.5–62.5	− 2.03	.05
Number of people in home	3.73 (1.01)	2–6	4.3 (1.87)	2–9	− 1.24	.23
Infant age at stool sample collection (days)	383.03 (18.06)	322–429	387.9 (17.63)	368–450	− 0.97	.33

	N	% of sample	N	% of sample	Chi-square value	*P*-value
Siblings (yes/no)	15 yes	44	6 yes	30	0.84	.36
Method of delivery	24 vaginal	71	15 vaginal	75	> 0.001	1
Presence of pet (yes/no)	8 yes	24	6 yes	30	> 0.001	1
Still breastfeeding at 12 months (yes/no)	14 yes	41	11 yes	55	0.49	.48
**Demographics**						
**Infant race**					1.95	.58
Other/two or more races	14	41	8	40		
White	8	24	8	40		
Black/African American	7	21	2	10		
Asian	3	9	2	10		
Not specified	2	6	0	0		
**Infant ethnicity**					> 0.001	1
Hispanic/Latine	14	41	8	40		
Not Hispanic/Latine	18	53	12	60		
Not specified	2	6	0	0		
**Infant sex**					0.17	.68
Male	22	65	11	55		
Female	12	35	9	45		

We performed a series of t-tests and chi-square tests to examine differences between groups on the social and demographic measures featured in Table 1. The groups did not differ significantly on measures of socioeconomic status, racial demographics, infant sex, caregiver mental health, material hardship, or lifestyle factors (Table 1). The only group difference that emerged was a modestly higher average daily fat intake in the pandemic group relative to the pre-pandemic group (t = − 2.03, *p* = 0.05). Following up on this finding, we tested correlations between average daily fat intake and our measures of gut microbiota alpha diversity, to examine whether this measure explained some variance in gut microbiota diversity in our sample. Importantly, we saw that average daily fat intake was negatively associated with Chao1 diversity across the whole sample (r = − 0.42, *p* = 0.01, data not shown). Thus, we control for fat intake in subsequent analyses. We also included infant sex, infant method of delivery (cesarean section or vaginal), and whether the infant was still breastfeeding at sample collection (yes/no) as covariates in all analyses based on prior empirical and theoretical associations with gut microbiome composition^6,18,27^ and a subsequent data-driven approach to covariate selection (see methods for more details). Results without covariates are presented in the supplement.

### Gut microbiome alpha diversity by pandemic group

**Figure 2:**
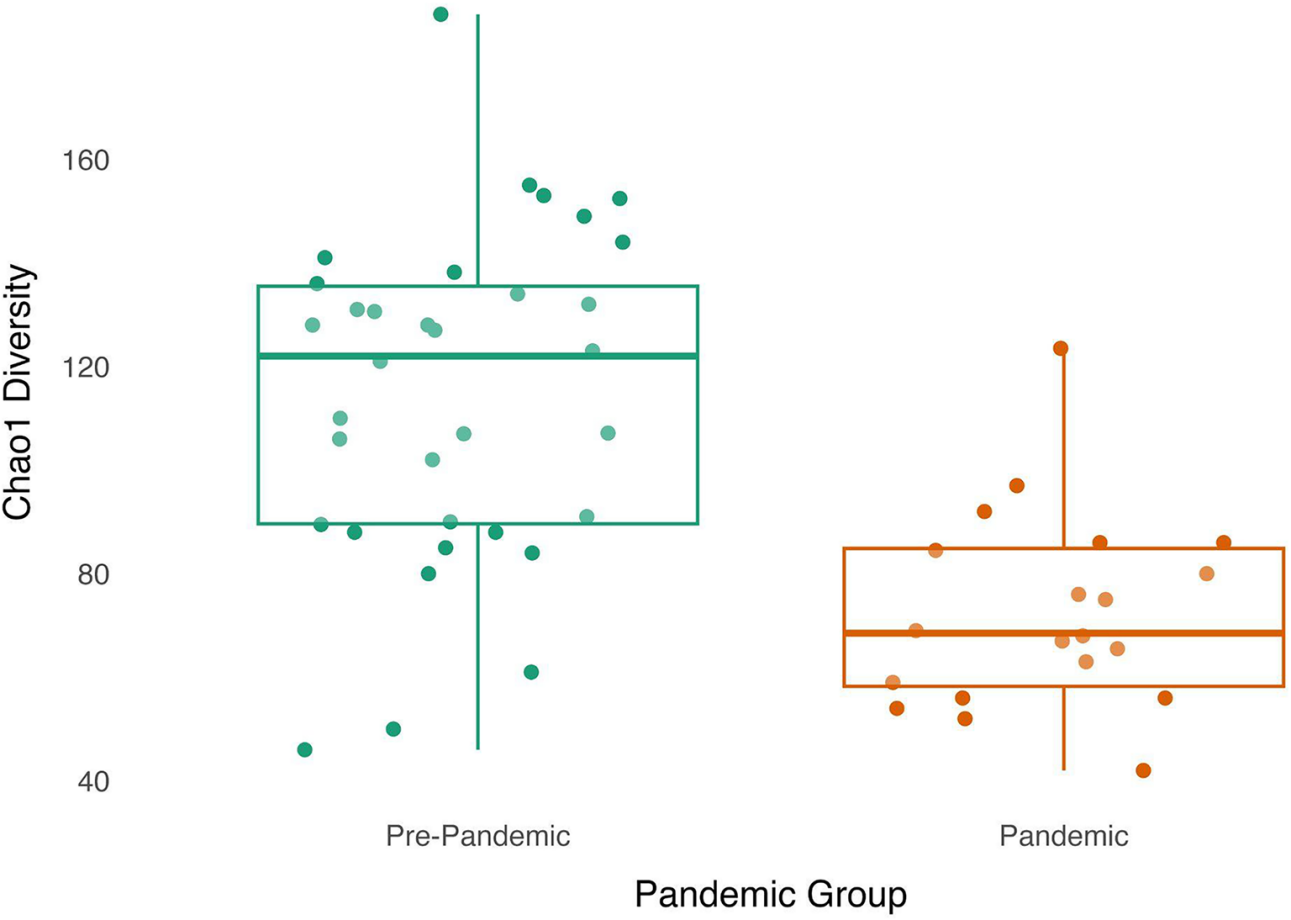
Chao1 diversity was significantly lower in the pandemic group. Infants sampled during the pandemic (N = 20; orange box plot and dots) had significantly lower alpha diversity, measured via the Chao1 index, than infants sampled before the pandemic (N = 34; green box plot and dots). Dots have been jittered along the x-axis to increase visibility of individual data points. Boxplot represents the median (line in the middle of the box), upper 25% quantile (top of the box), lower 25% quantile (bottom of the box), upper 25% quantile minus 1.5 times the interquartile range (upper whisker), and lower 25% quantile minus 1.5 times the interquartile range (lower whisker) Chao1 diversity values.

Shannon diversity did not significantly differ between pre-pandemic and pandemic samples ($\beta$ =− 0.15, *p* = 0.19; see Supplementary Materials Figure S1). In contrast, Chao1 diversity was significantly lower within pandemic samples compared to pre-pandemic samples ($\beta$=− 0.51, *p* < 0.001), see Fig. 2.

### Alpha diversity of gut microbiota by days since the start of the pandemic among the pandemic group

**Figure 3:**
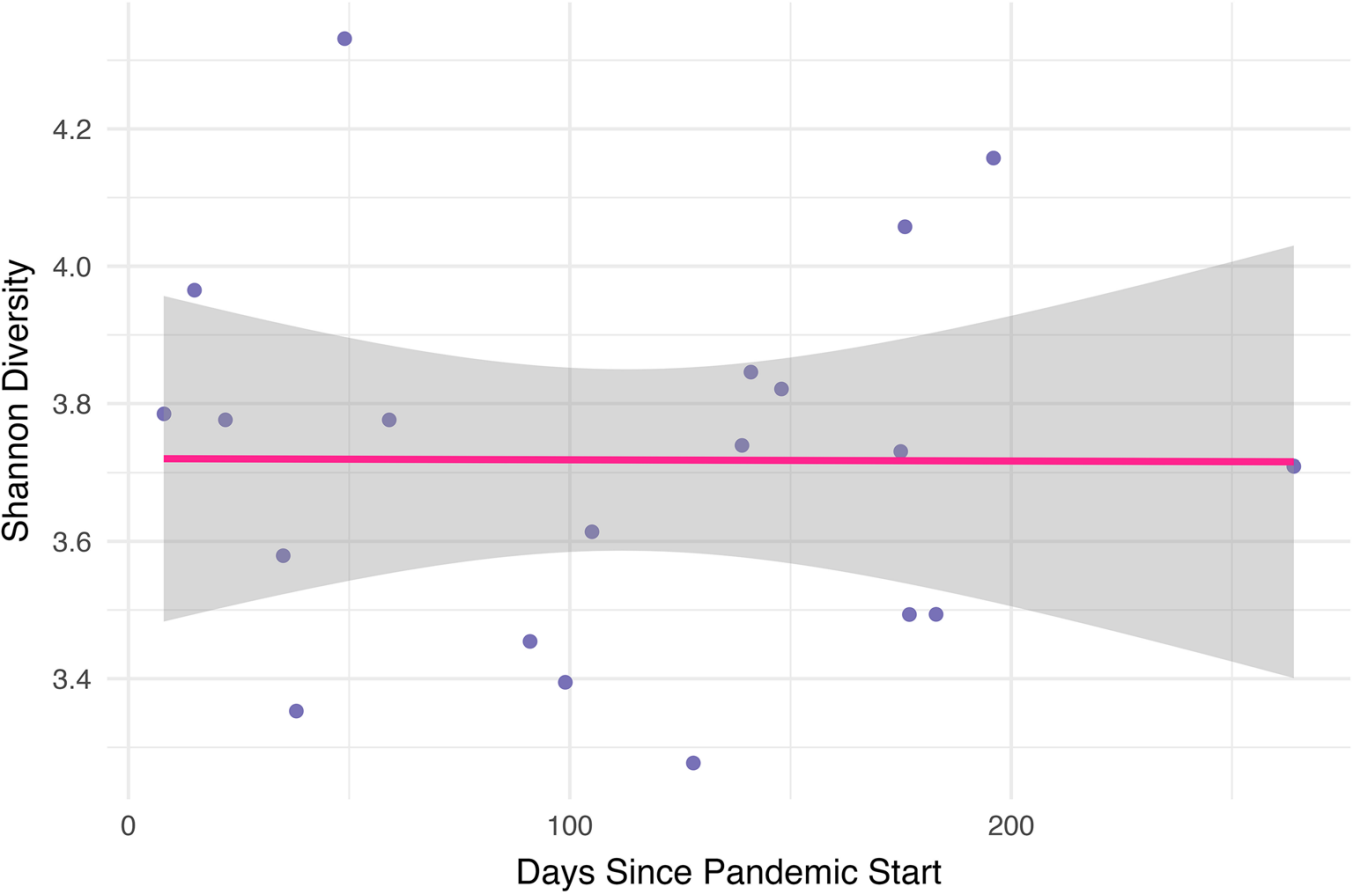
Shannon diversity by days since the start of the pandemic. Within the pandemic group, we found no significant correlation between Shannon diversity and days since the start of the pandemic.

**Figure 4:**
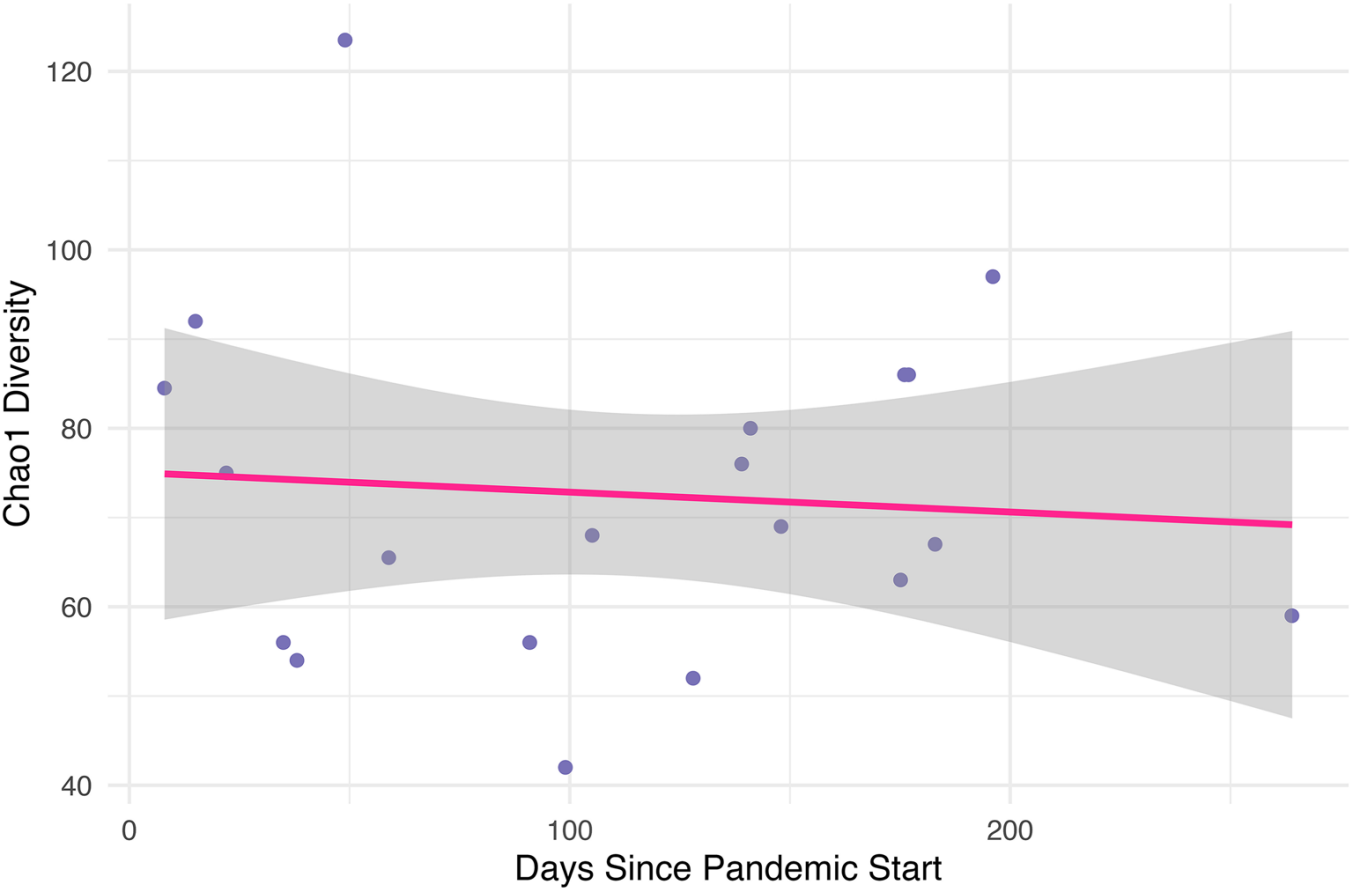
Chao1 diversity by days since the start of the pandemic. Within the pandemic group, we found no significant correlation between Chao1 diversity and days since the start of the pandemic.

Within the pandemic group, we calculated the number of days between the start of the pandemic and sample collection (range = 8–256 days). There were no significant associations between collection date and either Shannon (r = − 0.004, *p* = 0.98) or Chao1 (r = − 0.084, *p* = 0.72) diversity (Figs. 3 and 4, respectively).

### Gut microbiome beta diversity by pandemic group

**Figure 5:**
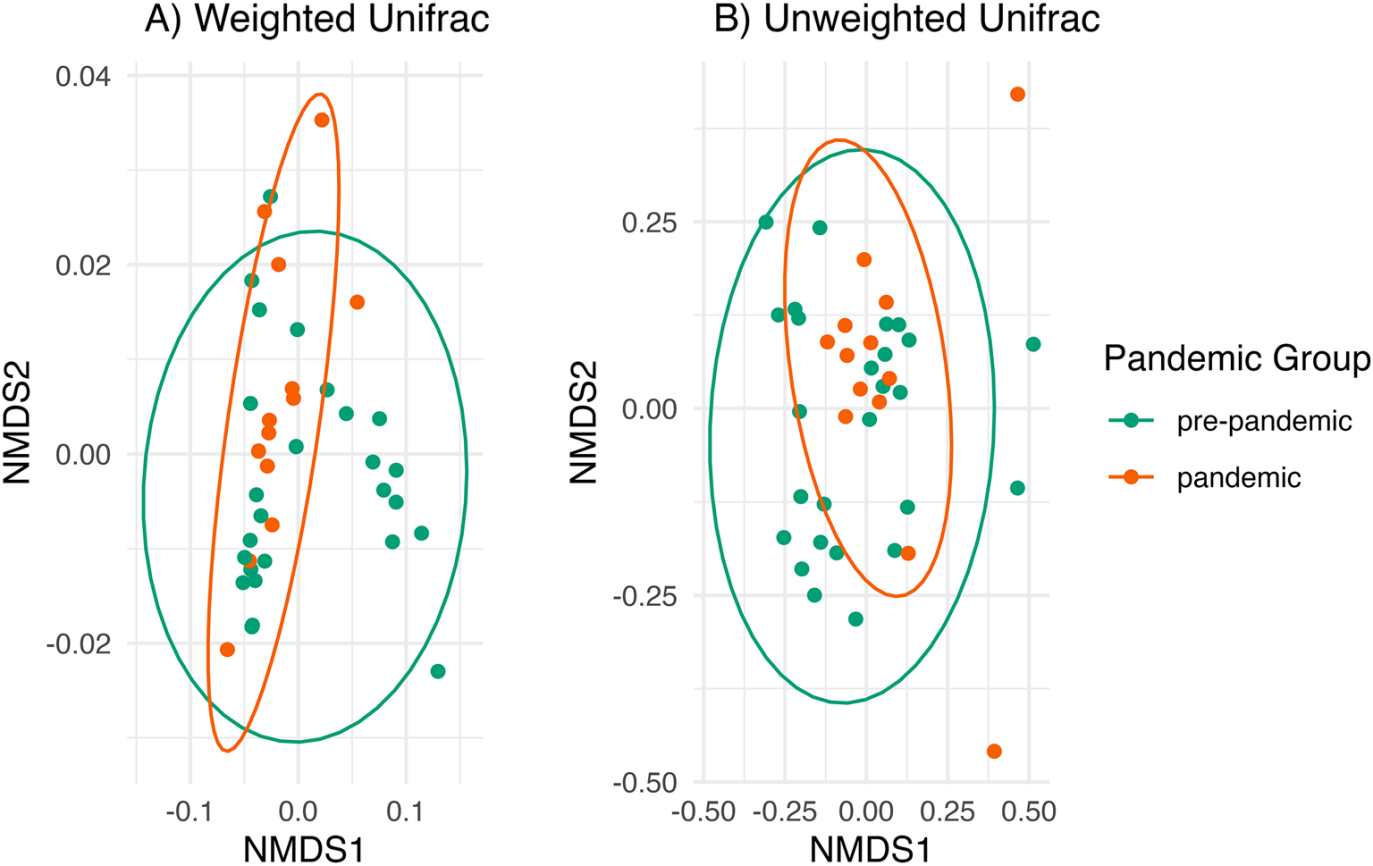
Beta diversity by pandemic group. (**A**) Pandemic group had significantly different gut microbial richness measured with Unweighted Unifrac distance, which can be seen in that the pre-pandemic samples (N = 24; green) are more spread out along the x-axis (NMDS1) than the pandemic samples (N = 13; orange). (**B**) Pandemic groups violate homogeneity of dispersion using weighted Unifrac distance: the pandemic samples are less dispersed (more tightly packed together) than the pre-pandemic samples. Each dot represents a participant; the closer the dots are to each other, the more similar their microbial communities. There are N = 13 pandemic samples and 24 pre-pandemic samples as beta diversity analyses were performed on the subsample with complete data on all covariates. Ellipses for each pandemic group are based on the 95% confidence level for a multivariate *t*-distribution.

Homogeneity of dispersion across pandemic groups, a necessary assumption for permutational anova beta diversity analyses, was violated using weighted Unifrac distance (*F*(1,37) = 5.63, *p* = 0.02): the pandemic samples appear to be less dispersed than the pre-pandemic samples using weighted Unifrac (Fig. 5). Using unweighted Unifrac, homogeneity of dispersion across pandemic groups was not violated (*F*(1,37) = 2.33, *p* = 0.14). Thus, we tested beta diversity by pandemic group using unweighted Unifrac distance, which measures microbial richness (presence/absence of each taxon). We found that the pre-pandemic and pandemic groups had significantly different gut microbial richness, (*F*(5, 33) = 1.69, *p* = 0.006, R^2^ = 0.04).

### Differential abundance of gut microbiome by pandemic group

#### Targeted analysis: family-level

**Figure 6:**
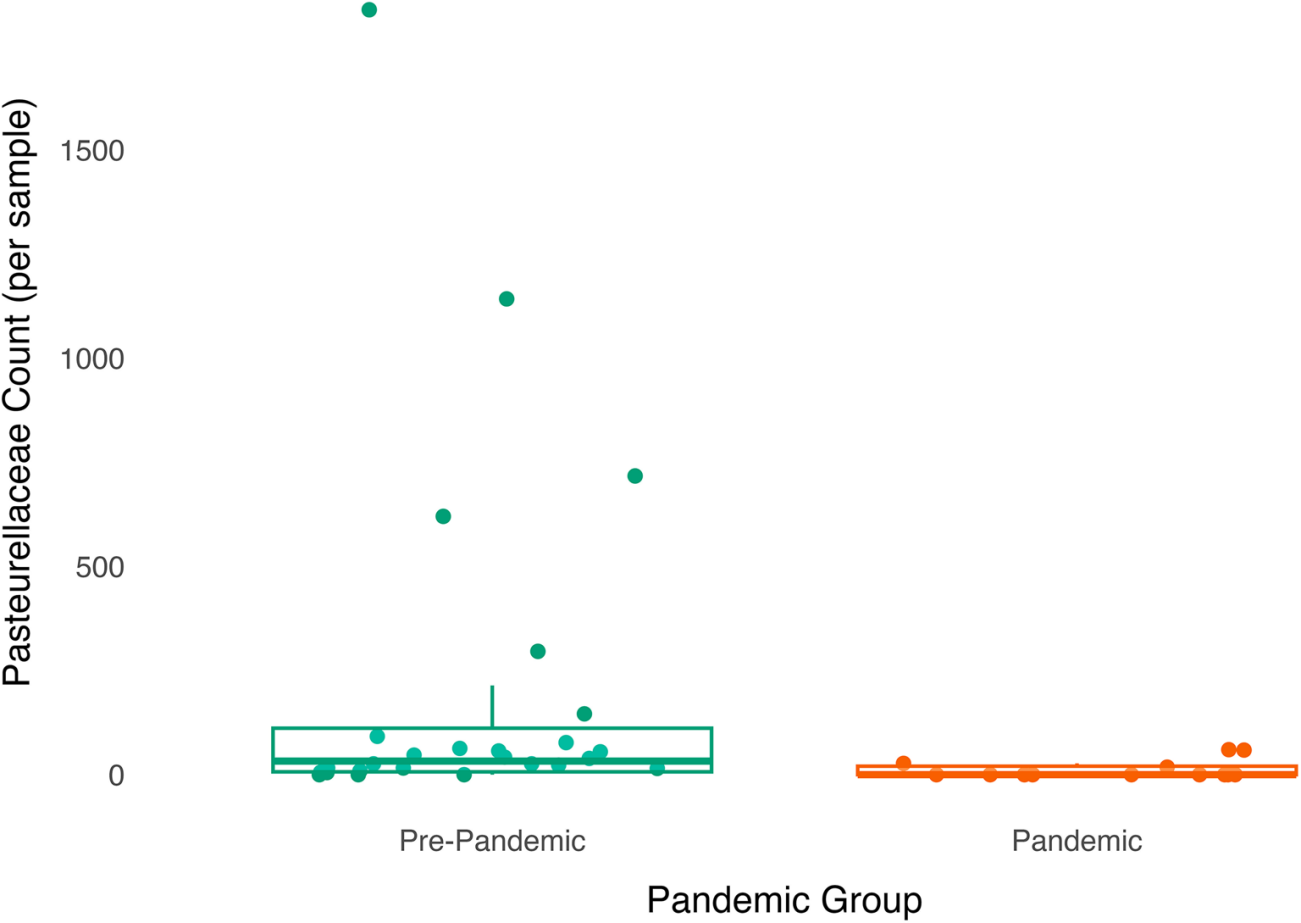
Abundance of taxa from the Pasteurellacae family in the gut microbiome is lower among infants sampled during the pandemic than infants sampled pre-pandemic. Dots (green are pre-pandemic samples, orange are pandemic samples) have been jittered along the x-axis to increase visibility of individual data points. Boxplot represents the median (line in the middle of the box), upper 25% quantile (top of the box), lower 25% quantile (bottom of the box), upper 25% quantile minus 1.5 times the interquartile range (upper whisker), and lower 25% quantile minus 1.5 times the interquartile range (lower whisker) abundance values. N = 13 pandemic samples and 24 pre-pandemic samples as differential abundance analyses were performed on the subsample with complete data on all covariates.

Compared to the pre-pandemic samples, pandemic samples had a lower relative abundance of Pasteurellaceae ($\beta$ = 2.52, SE = 0.88, *p* = 0.007, *q* = 0.14; Fig. 6).

#### Exploratory analysis: species and genus-levels

**Figure 7:**
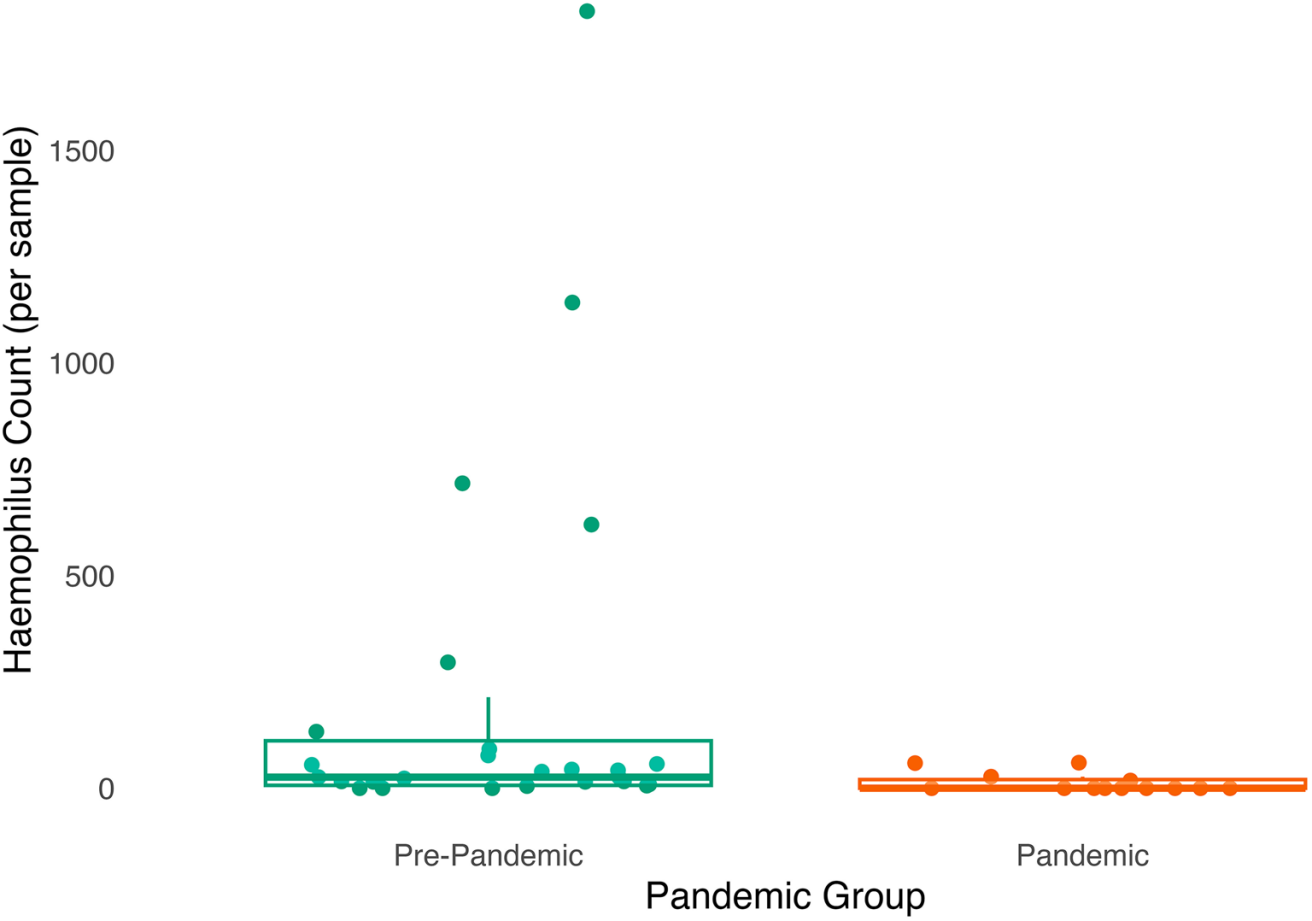
Abundance of taxa from the *Haemophilus* genus is lower in the gut microbiome of infants sampled during compared to before the pandemic. Dots (green are pre-pandemic samples, orange are pandemic samples) have been jittered along the x-axis to increase visibility of individual data points. Boxplot represents the median (line in the middle of the box), upper 25% quantile (top of the box), lower 25% quantile (bottom of the box), upper 25% quantile minus 1.5 times the interquartile range (upper whisker), and lower 25% quantile minus 1.5 times the interquartile range (lower whisker) abundance values. N = 13 pandemic samples and 24 pre-pandemic samples as differential abundance analyses were performed on the subsample with complete data on all covariates.

Taking an exploratory approach, no species were differentially abundant as a function of pandemic group. Taxa from the *Haemophilus* ($\beta$ = 2.31, SE = 0.87, *p* = 0.01, *q* = 0.13) genus were significantly less abundant in the gut microbiome of infants sampled during the pandemic compared to before the pandemic (Fig. 7).

### Differential abundance of gut microbiota by days since the start of the pandemic among the pandemic group

#### Targeted analysis: family-level

Abundance of taxa from the Bifidobacteriaceae family was negatively associated with days since the start of the pandemic ($\beta$ = − 0.56, SE = 0.25, *p* = 0.038, *q* = 0.23).

#### Exploratory analysis: species and genus-levels

Taking an exploratory approach, no taxa were differentially abundant according to days since the start of the pandemic on the species or genus levels.

### Sensitivity analyses excluding infants with recent antibiotics exposure

Excluding two infants with recent antibiotics exposure, Chao1 diversity was significantly lower within pandemic samples compared to pre-pandemic samples ($\beta \hspace{0.17em}$= − 0.49, *p* < 0.001); Shannon diversity did not differ significantly between the pandemic groups ($\beta \hspace{0.17em}$= − 0.14, *p* = 0.24). See supplementary materials for more information.

## Discussion

This study reports differences in gut microbiome composition within a cohort of 12-month old infants who were either sampled before the start of the COVID-19 pandemic or during the first 9 months of the pandemic. Specifically, we found that pandemic samples had lower alpha diversity of the microbiome, significantly different gut microbial richness based on beta diversity analyses, lower abundance of Pasteurellaceae taxa, and lower abundance of *Haemophilus* taxa than pre-pandemic samples. These results suggest that pandemic-related environmental disruptions had an impact on community-level taxonomic diversity of the early life gut microbiome as well as abundance of specific members of the gut bacterial community.

This study is the first to investigate potential impacts of the pandemic on the developing gut microbiome, and contributes to a larger body of research that has documented relations between early environmental experiences and taxonomic diversity of the gut microbiome. In our study, lower alpha diversity was observed in the pandemic group, and the two groups had significantly different gut microbial richness. Other experiences of physical and social disruption, arguably similar to those that were instigated by the pandemic, including exposure to antibiotics and to caregiving adversity in the first year of life, have been associated with lower alpha diversity and significant differences in beta diversity in the developing gut microbiome^3,28,29^. Pandemic groups in our sample did not differ on measures of maternal perceived stress or mental health, potentially indicating that pandemic-related differences in the gut microbiome were driven by factors distinct from maternal psychosocial stress.

While speculation on the potential implications of differences in gut microbiome composition measured at a single time point for long-term health should be interpreted with caution, alpha diversity has been linked to health outcomes across the lifespan. In adulthood, lower alpha diversity is typically associated with poorer health outcomes^30^. Findings on alpha diversity and child health have been somewhat mixed: some studies have found lower alpha diversity concurrently or prospectively linked to conditions including asthma, atopic dermatitis, food allergy, and type 1 diabetes, while others have found no associations^18,31–34^. Importantly, there is no established reference for the “typical” or “optimal” level of alpha diversity at any point in development, and the gut microbiome is continuously changing in response to environmental inputs in early life; some of those changes may be transient or unrelated to future outcomes. Longitudinal studies that follow children who experienced the pandemic during early life are needed to understand the implications of pandemic-related impacts on the gut microbiome for health and development.

In addition to differences in community-level taxonomic diversity across pandemic groups, we also found several differences on the individual taxa level: lower abundance of taxa from the Pasteurellaceae family and the *Haemophilus* genus within Pasteurellaceae among infants sampled during the pandemic relative to before the pandemic. Lower *Haemophilus* abundance has also been associated with more frequent use of cleaning/disinfectant products in infants and young children^4,35^, though Pasteurellaceae and *Haemophilus* abundance was found to be higher in infants cared for at home compared to those in childcare^8,36^, and those in childcare are presumably exposed to a larger number of same-age peer children as well as more stringent cleaning protocols. Taken together, our findings in combination with those from prior studies suggest that the lower abundance of Pasteurellaceae and *Haemophilus* observed in infants sampled during compared to before the pandemic may be due to more frequent cleaning during the early waves of the pandemic, rather than differences in social contact patterns.

Additional exploratory analyses conducted within the pandemic group addressed whether gut microbiome features differed according to the number of days since the pandemic began. Though we found differences in taxa abundance associated with days since pandemic onset, these differences were not within the same taxa as those found to be differentially abundant across pandemic groups, suggesting that pandemic-related effects on the gut microbiome did not differ according to length of time families had spent in the pandemic. Instead, we speculate that gut microbiome changes due to the pandemic occurred relatively quickly and were sustained, rather than accumulating gradually over time. However, due to the smaller sample size for this analysis, and changes in recommended public health precautions across the time period when pandemic samples were collected (e.g., increased outdoor socializing opportunities during the summer due to warmer weather), the conclusions we can draw from this analysis are limited.

Strengths of this study include a socioeconomically diverse sample and natural experiment design. Because the groups of families sampled before versus during the pandemic were demographically similar, a relatively well-controlled comparison could be made between pandemic groups. However, we were only able to include a small sample of infants (n = 54) in the study, limiting our power to detect subtle and individual taxa-level differences within the gut microbiome across pandemic groups via differential abundance analyses, particularly within rare taxa. Indeed, Chao1 diversity (found to be lower after the start of the pandemic) factors rare taxa into measurement of global gut microbial diversity, while Shannon diversity does not. Similarly, unweighted Unifrac distance (found to differ by pandemic group) allows for rare taxa to be influential, while weighted Unifrac does not. As such, it may be the case that the group differences we observed with Chao1 diversity and unweighted Unifrac distance are at least partially driven by rare taxa that we are not powered to detect with differential abundance analyses in a sample of this size. As all families who participated were living in New York City, an urban context severely impacted by COVID-19 during the first year of the pandemic compared to the rest of the United States, the generalizability of our findings are limited. It is possible that geographic differences in environmental changes caused by the pandemic could have had different impacts on the early life gut microbiome, and/or that impacts of the pandemic on the gut microbiome could differ according to regional differences in gut microbiome composition^37^.

Importantly, this study was not able to directly measure how every aspect of families’ environments changed as a result of the pandemic, and thus was limited in our examination of which environmental changes may have driven the observed differences in gut microbiome composition across groups. Though we have no reason to suspect families differed across groups on parameters such as adherence to social distancing directives, we do not know how or to what degree their lives changed during the pandemic. While we were not powered to examine how social determinants may be related to effects of the pandemic on infants’ gut microbiomes, given the well-known inequitable impact of the pandemic within the United States according to gradients of social disadvantage, further research should examine how dimensions of social advantage may influence the relationship between pandemic-related environmental disruptions and infants’ biophysiological development.

## Conclusions

It is rare to have a natural experiment, such as that afforded by the dramatic environmental changes that occurred when the COVID-19 pandemic began, in which to examine environmental influences on the early life gut microbiome. Overall, evidence from this study suggests that pandemic-related disruptions to infants’ environments had an impact on their gut microbiomes, with lower alpha diversity, differences in beta diversity, and lower abundance of several taxa seen in infants sampled during the first year of the pandemic compared to before the pandemic. Further research is needed to understand if differences in gut microbiome composition have persisted for these children, how pandemic disruptions may have influenced the gut microbiome of children at different developmental stages, and the long-term implications of these microbiome differences for children’s health-related outcomes. Results can inform our scientific understanding of microbiome plasticity to changing environmental conditions and contribute to a growing body of research on child development during the pandemic.

## Methods

### Participants

One-hundred families from the greater New York City area were enrolled into a longitudinal study examining early neurocognitive development. Infants who were born at or after 36 weeks of gestation and had no history of neurological or developmental delays were included.

### Procedures

The first wave of data collection, when infants were 3-months of age, took place between April 2018 and December 2019. At 3 months caregivers completed a number of demographic and mental health surveys. Stool samples were collected when infants were 12 months old, between December 2018 and December 2020. At 12 months, all caregivers completed a similar series of surveys over the phone, including a food diary for themselves and their infant, and provided an infant stool sample via mail. All families were contacted to provide a stool sample and 54 families returned usable samples. Thirty-four caregivers provided infant stool samples before the start of the pandemic (pre-pandemic group), and 20 provided samples after the start of the pandemic (pandemic group). March 22, 2020 was used as the date for the start of the COVID-19 pandemic in this sample, as that was the date that all non-essential businesses closed and group social gatherings were prohibited in New York State under Executive Order 202.8^38^. All procedures were approved by the Institutional Review Board at New York University and performed in accordance with relevant guidelines, and informed consent was obtained from primary caregivers for themselves and the infant. A timeline of data collection can be seen in Fig. 1.

### Measures

#### Stool sample collection and processing

When infants were 12 months old, caregivers received a stool sample collection kit in the mail (OMNIgene.Gut; DNA Genotek), instructions, a brief survey on the date/time of collection, and a prepaid shipping envelope to return the sample to the study team. Samples were subject to 16 s RNA sequencing (see below for more information). After initial data processing steps, functions from the phyloseq package v1.28.0 were used to generate microbial alpha diversity estimates in R 3.6.1 (2). We focused on the Chao1 index, which examines the number of bacteria taxa present in the sample, and the Shannon index, which takes into account both the number of taxa and the evenness of the distribution of taxa.

Raw sequencing reads were adapter-trimmed and demultiplexed after FASTQ conversion in BaseSpace (Illumina). Divisive amplicon denoising algorithm version 2 (DADA2 1.12.1) was used to trim, dereplicate, and filter chimeric sequences before generating amplicon sequence variant (ASV) Table 1. Based on the quality score profiles of sequencing reads, forward reads were truncated at 250 bp and reverse reads were truncated at 240 bp prior to merging, ambiguities in the overlap region were not allowed, and default parameters were otherwise applied in the R *dada2*package filterAndTrim() function [truncLen = c(250,240); trimLeft = c(5,5), maxN = 0, maxEE = c(2,2), truncQ = 2]. After dereplication and merging of reads, chimeric reads were identified by consensus across samples using the DADA2 function removeBimeraDenovo(). All samples passed the imposed minimum of 7500 reads after quality filtering for inclusion in this analysis. The MAFFT and FastTree modules in QIIME2 were used to generate a phylogenetic tree of all ASV sequences.

#### Potential covariates

##### Demographic and lifestyle questionnaire

Questions regarding infant race and ethnicity, infant sex, caregiver education, family income, number of siblings to the enrolled infant, household material hardship, and breastfeeding were solicited from caregivers when infants were 3 months. At 12 months, caregivers reported on method of delivery at birth, breastfeeding, infant antibiotic/probiotic use, exposure to pets in the home, and infant diet. See supplemental information for full description of these measures.

##### Caregiver Mental Health

Caregivers completed the Edinburgh postnatal depression scale (EPDS), the perceived stress scale (PSS), and the State/Trait Anxiety Inventory when infants were 3 months old. At 12 months, they completed the PSS and the patient health questionnaire—9 (PHQ-9)^39–42^. Full descriptions of these measures can be found in the supplementary materials.

### Statistical analyses

For all analyses, we designated a significance threshold of *p* < 0.05. All analyses were conducted using R version 4.2.3^43^**.**

#### Covariate selection

We identified potential covariates from variables in our dataset a priori based on prior research on factors known to impact the gut microbiome at 12 months of age: infant age at stool sample collection, infant sex, birth mode (cesarean section or vaginal), number of people in the infant’s household, pets in the household (yes/no), siblings in the household (yes/no), whether the infant was still breastfeeding (yes/no) at sample collection, and infant diet variables including average daily protein, fiber, and fat consumption^6,18,27,44,45^. Given our dataset’s small sample size, we then took a data-driven approach to select a subset of covariates from this list that were most likely to affect our results. We first tested for group differences on each potential covariate across pandemic groups, including any variables that significantly differed across groups. We then tested whether each variable explained significant variance in gut microbiome alpha diversity. Within the subset of variables that did explain significant variance, we tested pairwise associations (Pearson’s correlation, *t*-test, or chi-square test depending on variable types). If two variables were significantly related, we included both only if the related variables each explained significant additional variance in gut microbiome alpha diversity (determined via significance of an ANOVA comparing a regression model containing only one of the covariates predicting alpha diversity to a model containing both covariates). If each variable did not explain significant additional variance in alpha diversity, we included the variable of the two with less missing data, in order to maximize our sample size. Following this process, we arrived at the following set of covariates which are included in all analyses examining both the pre-pandemic and during pandemic groups: average fat intake, child sex, still breastfeeding at sample collection, and birth mode. Due to the small sample size of the during pandemic group (N = 20), analyses restricted to this group did not include any covariates.

#### Missing data handling

Among our sample of infants who donated a usable stool sample for analysis, 14 (25.9%) were missing data on average fat intake, none were missing data on child sex, none were missing data on still breastfeeding at sample collection, and 1 (1.8%) was missing data on birth mode.

For alpha diversity analyses, we used full information maximum likelihood using the lavaan R package^46^ to treat missing values. For beta diversity and differential abundance analyses, which are microbiome-specific analysis techniques that lack a more sophisticated missing data handling method (e.g., multiple imputation) that can be implemented with currently available software and methodology without requiring extensive novel code^47^, we used the subsample of N = 39 infants (26 in the pre-pandemic, 13 in the during pandemic group) with complete data on all covariates.

#### Alpha diversity

We conducted a series of t-tests and chi-square tests to compare the pre-pandemic and pandemic groups on demographic and environmental characteristics. Measures that differed significantly between groups were included as covariates in subsequent analyses. T-tests and multiple linear regressions were used to examine whether alpha diversity differed between pandemic groups.

#### Beta diversity

Beta diversity was calculated between infant gut microbiomes grouped according to pandemic group. Phylogeny-based unweighted Unifrac (taxa are not weighted by abundance) and weighted Unifrac (taxa are weighted based on their abundance; thus, this metric is less driven by rare taxa compared to unweighted Unifrac) distances were calculated^48^. To test for significant differences across the groups, we used the permutational multivariate analysis of variance (PERMANOVA), an analog to MANOVA designed for partitioning distance matrices according to various sources of variation, using the *adonis2()* function from the vegan R package^49^. Since this test is sensitive to heterogeneity of data dispersion across groups, we also performed an analysis testing multivariate homogeneity of dispersion (PERMDISP)^50^ using the *betadisper()* function, also from the vegan R package. For the PERMANOVA and PERMDISP tests, the number of permutations was set to 9999. Beta diversity results were visualized with non-metric multidimensional scaling (NMDS)^51^ using the *ordinate()* function from the phyloseq R package.

#### Differential abundance

To examine differential abundance, we used Microbiome Multivariable Associations with Linear Models v2 (MaAsLin 2) R package v1.7.3 with default model type, transformation, and normalization parameters^52^. MaAsLin 2 derives the relative abundance of each taxon per sample by dividing the count for each taxon by the total count for all taxa within the sample. To maximize power for differential abundance analyses, we filtered our dataset to only examine taxa that had non-zero abundance in at least 50% of samples (20 species and 22 genera met this criterion). *P*-values were corrected for multiple comparisons within sets of analyses that examined abundance on the species-level (20 comparisons), on the genus-level (22 comparisons), and the family level (8 comparisons), with a *q*-value threshold for significance of 0.25 as has been used in prior work and recommended for biomarker discovery approaches^53^. Next, we performed follow-up analyses within the pandemic group to see whether any gut microbiome features would be associated with the number of days since the pandemic began using the same methods as above.

### Supplementary Information

Supplementary Information.

## Data Availability

De-identified data is available in the Open Science Framework project page: https://osf.io/qgw7x/.
