# Supplementary material for: A comparison of the infant gut microbiome before versus after the start of the covid-19 pandemic
Source: Sci Rep. 2023 Aug 16;13:13289. doi: 10.1038/s41598-023-40102-y (PMC10432475; doi:10.1038/s41598-023-40102-y)
Supplement: Supplementary file 1 — Supplementary Information. [file 41598_2023_40102_MOESM1_ESM.docx]

**Supplemental Methods**

**Measures**

***Demographic and Lifestyle Questionnaires***

When infants were 3 months old, caregivers completed a series of questionnaires assessing caregiver and child race and ethnicity , child sex, caregiver education, total family income, and the number of people supported by the family income. Caregivers also reported on the extent to which they breastfeed versus formula feed their infant. Finally, caregivers completed the Material Deprivation Scale[^1^](https://paperpile.com/c/6RwVPe/uyxY), which assessed the extent to which the family has struggled to meet basic material needs such as food, housing, utilities, or medical care.

At 12 months of age, caregivers again reported on breastfeeding, as well as if the infant had taken any antibiotic, probiotic, or antifungal medications or supplements in the preceding month and the type and dose, if applicable. Caregivers also provided information on if the child was raised in a home with a pet, what the method of delivery was when the infant was born (vaginal vs. cesarean), if the infant was born outside of the United States or had spent four months or more outside of the United States. Finally, caregivers completed two days of food diaries for themselves and the infant, where they listed everything they and the infant ate and drank for two days. These data were used to calculate daily averages for carbohydrates, fats, and protein.

***Caregiver Mental Health***

**Edinburgh postnatal depression scale (EPDS).** Caregivers completed the 10-item EPDS [^2^](https://paperpile.com/c/6RwVPe/UAHO) when infants were 3 months old to assess common symptoms of postpartum depression experienced during the previous week. Some sample items include: “I have been able to laugh and see the positive side of things as much as I used to,” “I have been so unhappy that I have had difficulty sleeping,” and “Things have been piling up and I haven't been able to keep up.”

**Stait-trait anxiety inventory (STAI).** The STAI is an anxiety questionnaire that assesses current feelings of anxiety (state) and overall anxiety (trait). Caregivers complete the STAI when infants are 3 months old. The state anxiety scale assesses the degree to which participants are experiencing symptoms of anxiety in the moment they are completing the questionnaire. The item prompt is: “Give the answer which seems to describe your PRESENT feelings best.” Sample items include: “I feel calm,” “I am jittery,” “I feel satisfied,” and “I feel indecisive.” The trait anxiety scale assesses the degree to which participants experience symptoms of anxiety in general. The prompt is: “Give the answer which seems to describe how you GENERALLY feel.” Sample items include: “I wish I could be as happy as others seem to be,” “I feel cool, calm, and collected,” “I lack self-confidence,” and “I am a steady person.” Answer options for both scales consist of “Not at all,” “Somewhat,” “Moderately so,” and “Very much so.”

**Perceived stress scale (PSS).** The PSS is a 10-item parent-report measure of self-reported stress levels over the preceding month. The primary caregiver completed the PSS when the child was 3 months old and again when the child was 12 months old. Items are rated on a scale from 0-4, where 0 corresponds to “Never” and 4 to “Very Often.” Sample items include: “In the past month, how often have you been upset because of something that happened unexpectedly,” “In the last month, how often have you found that you could not cope with all the things that you had to do,” and “In the last month, how often have you felt nervous and stressed?” Total scores are calculated as the sum of all answers and can range from 0-40.

**Patient health questionnaire depression screening (PHQ-9).** The PHQ-9 [^3^](https://paperpile.com/c/6RwVPe/U2tt) is a 9-item scale completed by the primary caregiver when the infant is 12 months old assessing symptoms of depression. The questionnaire asks to what extent the participant has been bothered by a series of problems within the last two weeks; some sample items include: “Feeling bad about yourself—or that you are a failure or have let yourself or your family down,” “Little interest or pleasure in doing things,” or “Feeling tired or having little energy.” Answer options include: “Not at all,” “Several days,” “More than half the days,” or “Nearly every day.” Answers are coded from 0-3, respectively, and a total score is calculated as the sum of all answers for a possible range from 0-27.

**Statistical Analyses**

***Sensitivity Analyses***

We performed sensitivity analyses for alpha diversity excluding the two infants (one sampled during the pandemic, the other sampled pre-pandemic) within our sample who reported antibiotic use within a month preceding stool collection to determine whether those infants may have been driving results. As these two infants were missing data on other covariates, they were already excluded from our sample for beta diversity and differential abundance analyses.

**Supplemental Results**

**Gut microbiome alpha diversity by pandemic group without controlling for covariates**

Without controlling for covariates, Shannon diversity did not differ significantly between the pre-pandemic and pandemic groups (t=1.62, p=0.11; Figure S1). Chao1 diversity was significantly lower in the pandemic group compared to the pre-pandemic group (t=6.06, p<0.001; main text Figure 2).


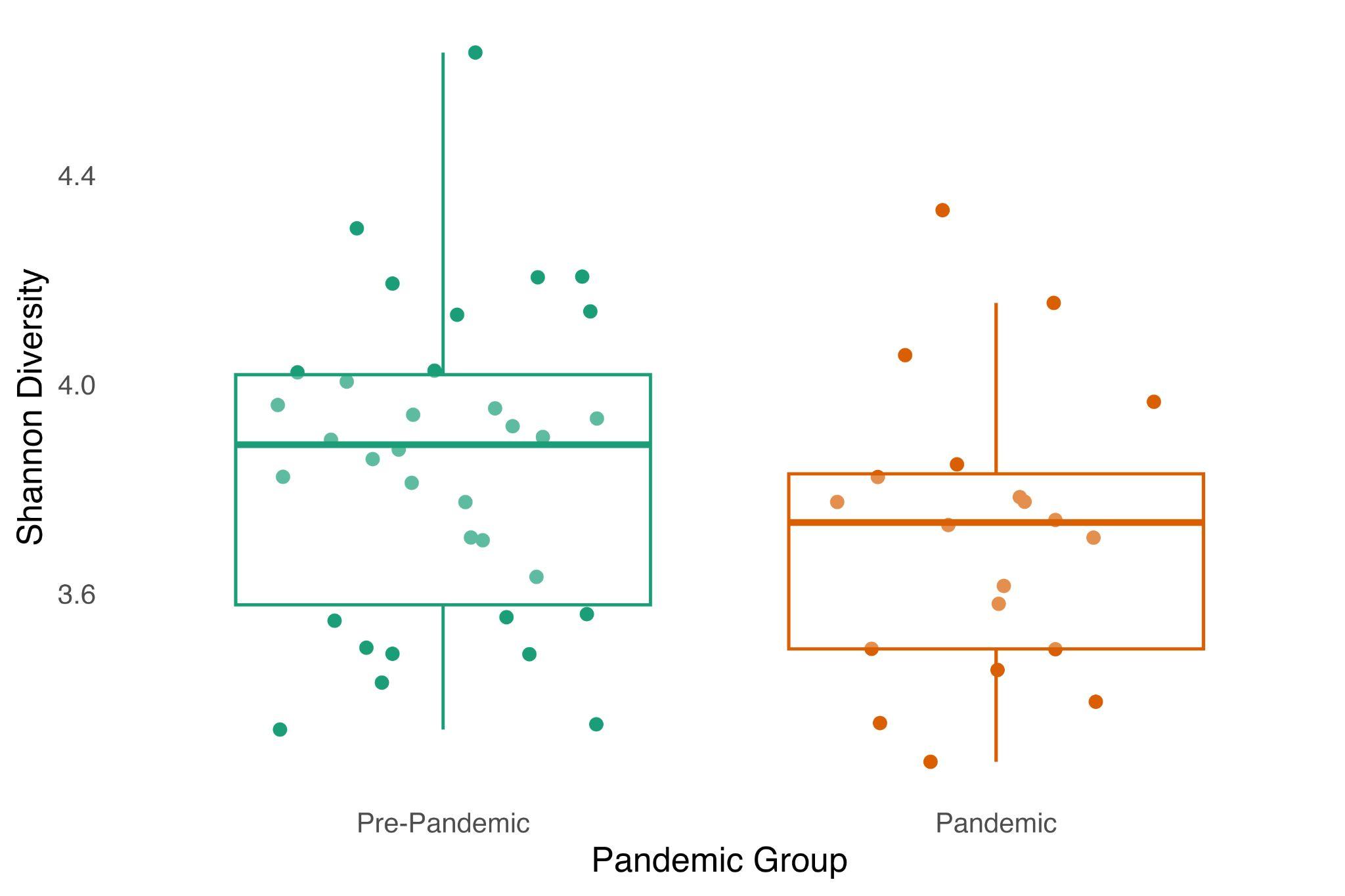


**Figure S1: Shannon diversity did not differ significantly between groups.** Infants sampled during the pandemic (N=20; orange box plot and dots) and infants samples before the pandemic (N=34; green box plot and dots) did not differ significantly on alpha diversity measures via the Shannon index. Dots have been jittered along the x-axis to increase visibility of individual data points. Boxplot represents the median (line in the middle of the box), upper 25% quantile (top of the box), lower 25% quantile (bottom of the box), upper 25% quantile minus 1.5 times the interquartile range (upper whisker), and lower 25% quantile minus 1.5 times the interquartile range (lower whisker) Chao1 diversity values.

**Gut microbiome beta diversity by pandemic group without controlling for covariates**

Without controlling for covariates, samples clustered significantly by pandemic group based on unweighted Unifrac distance (*F*(1, 37) = 1.65, *p* = .008, R^2 = .04); samples did not cluster significantly by pandemic group based on weighted Unifrac distance (*F*(1, 37) = 2.06, *p* = .13, R^2 = .05).

**Differential abundance of gut microbiome by pandemic group without controlling for covariates**

***Genus-level (whole microbiome)***

Taxa from the *Blautia* genus were significantly less abundant in the gut microbiota of infants sampled during the pandemic compared to those sampled before the pandemic; this finding was no longer significant after false-discovery rate correction ($\beta$ = 1.20, SE = 0.58, *p* = .042, *q* = .55).

***Species-level (whole microbiome)***

Without controlling for covariates, no species were differentially abundant as a function of pandemic group.

***Family-level (targeted analysis)***

Without controlling for covariates, taxa from the Pasteurellaceae family were significantly more abundant ($\beta$ = 1.85, SE= 0.83, *p* = .03*, q* = .12), and taxa from the Lactobacillaceae family were significantly less abundant ($\beta$ = -2.22, SE= 0.70, *p* = .002*, q* = .02), in the gut microbiome of infants sampled during the pandemic compared to before the pandemic.

**Supplemental References**

1. [Pilkauskas, N. V., Currie, J. & Garfinkel, I. The Great Recession, Public Transfers, and Material Hardship. *Soc. Serv. Rev.* **86**, 401–427 (2012).](http://paperpile.com/b/6RwVPe/uyxY)

2. [Cox, J. L., Holden, J. M. & Sagovsky, R. Detection of postnatal depression. Development of the 10-item Edinburgh Postnatal Depression Scale. *Br. J. Psychiatry* **150**, 782–786 (1987).](http://paperpile.com/b/6RwVPe/UAHO)

3. [Kroenke, K., Spitzer, R. L. & Williams, J. B. W. The PHQ-9: validity of a brief depression severity measure. *J. Gen. Intern. Med.* **16**, 606–613 (2001).](http://paperpile.com/b/6RwVPe/U2tt)
